# Evaluation of Autofluorescence in Identifying Parathyroid Glands by Measuring Parathyroid Hormone in Fine-Needle Biopsy Washings

**DOI:** 10.3389/fendo.2021.819503

**Published:** 2022-01-21

**Authors:** Zhen Liu, Run-sheng Ma, Jun-li Jia, Tao Wang, Dao-hong Zuo, De-tao Yin

**Affiliations:** ^1^ Department of Thyroid Surgery, The First Affiliated Hospital of Zhengzhou University, He’nan, China; ^2^ Department of Respiratory, The First Affiliated Hospital of Zhengzhou University, He’nan, China

**Keywords:** autofluorescence, parathyroid, thyroid surgery, nanocarbon, lymph node

## Abstract

**Background:**

Near-infrared autofluorescence imaging has potentially great value for assisting endocrine surgeons in identifying parathyroid glands and may dramatically change the surgical strategy of endocrine surgeons in thyroid surgery. This study is designed to objectively evaluate the role of near-infrared autofluorescence imaging in identifying parathyroid glands during thyroid surgery by measuring intraoperative parathyroid hormone in fine-needle aspiration biopsy washings.

**Methods:**

This study was conducted at a tertiary referral teaching hospital in China from February 2020 to June 2020. Patients undergoing total thyroidectomy with or without neck lymph node dissection were consecutively included. The surgeon used near-infrared autofluorescence imaging to identify parathyroid glands during thyroid surgery and confirmed suspicious parathyroid tissues by measuring their intraoperative parathyroid hormone. Nanocarbon was injected into the thyroid gland if the thyroid autofluorescence intensity was too strong. The sensitivity and accuracy of near-infrared autofluorescence imaging and vision for identifying parathyroid glands, and the difference in autofluorescence intensity in various tissues were the main outcomes.

**Results:**

Overall, 238 patients completed the trial. Based on the pathological and aIOPTH results, the sensitivity of near-infrared autofluorescence imaging for detecting parathyroid glands (568 of 596 parathyroid glands; 95.30%)was significantly higher than that of vision (517 of 596 parathyroid glands; 86.74%, *P*<.001). The accuracy of near-infrared autofluorescence imaging (764 of 841 tissues; 90.84%) was significantly higher than that of vision (567 of 841 tissues; 67.42%, *P*<.001) when the evaluations of certain tissues were inconsistent. There was a significant difference between the autofluorescence intensity of the parathyroid glands and that of the lymph nodes (74.19 ± 17.82 *vs* 33.97 ± 10.64, *P*<.001).

**Conclusion:**

The use of near-infrared autofluorescence imaging, along with intraoperative parathyroid hormone and nanocarbon for the identification of parathyroid glands in thyroid surgery may increase the number of confirmed parathyroid glands. Using near-infrared autofluorescence imaging can effectively distinguish lymph nodes and parathyroid glands during lymph node dissection.

## Introduction

Hypocalcemia after thyroid surgery is a problem that has long plagued endocrine surgeons. In a statistical analysis of 14,540 patients undergoing thyroidectomies in the United States from 2013 to 2015, 450 cases of severe postoperative hypocalcemia occurred (3.3% overall, 0.6% after partial, and 4.7% after subtotal or total thyroidectomy) (1). Severe postoperative hypocalcemia can be life-threatening and seriously impair quality of life. Accurate identification of parathyroid glands (PGs) is the first step to reduce postoperative hypocalcemia. At present, the recognition of PGs mainly depends on visual identification by the surgeon. However, due to their small size and unstable location, as well as the difficulty in distinguishing them in color and shape from lymph nodes and adipose tissue, highly subjective visual identification is often unreliable. Therefore, any technologies that might assist the visual identification of PGs can be of great clinical application value.

There have been a few reported studies relating to parathyroidectomy or thyroidectomy combined with autofluorescence, fluorescent methylene blue, 5-aminolevulinic acid, indocyanine green, optical coherence tomography, laser speckle contrast imaging, dynamic optical contrast imaging and Raman spectroscopy (2). These optical technologies could become complementary to the surgeon’s eyes and thus may reduce the hypocalcemia rate after thyroid surgery. Among them, near infrared-induced autofluorescence (NIRAF) technology based on the spontaneous auto fluorescent signal of the PG is most widely reported (3). Studies have shown that NIRAF can reduce the risk of postoperative hypocalcemia (4–6). However, reduction in hypocalcemia is only an indirect proof since it is also highly related to the further protection of recognized PGs.

The exact number of PGs identified by NIRAF is crucial to its clinical evaluation. In previous studies, NIRAF showed good sensitivity and specificity in the identification of PGs (7–9). However, all identified PGs during thyroid surgery need to be retained. Therefore, the pathological examination based on the study's purpose is against ethical requirements since it would cause permanent damage. Visual identification by the surgeon has been used in most trials as a confirmation method for PGs (10). The subjective visual identification itself lacks accuracy and is less persuasive to be used as the criterion. Although some of the trials used pathological examination to confirm PGs, the included patients mostly had parathyroid tumors or hyperparathyroidism (7). Many studies have shown that the NIRAF of diseased PGs is completely different from that of normal PGs, so it is better to exclude these patients in the study to explain the value of NIRAF in thyroid surgery (11, 12).

The intraoperative parathyroid aspiration for parathyroid hormone assay (aIOPTH) technology is considered to have high sensitivity (80%-100%) for the confirmation of PGs and has a high specificity close to 100% (13). Meanwhile, aIOPTH, as a minimally invasive examination method, causes minimal damage to PGs; consequently, evaluating suspected parathyroid tissues with aIOPTH completely meets the ethical requirements. Thus, aIOPTH is very suitable as an alternative to parathyroid pathology to evaluate the value of NIRAF.

This study was a large sample case series study including patients undergoing thyroidectomies. The purpose was to use aIOPTH to evaluate the value of NIRAF in thyroid surgery. We introduced several new methods to improve the effect of NIRAF during the study and tried to find a new strategy using NIRAF and aIOPTH to improve the identification of PGs.

## Methods

The study was approved by the institutional review board of the First Affiliated Hospital of Zhengzhou University (2019-KY-283) and registered on Chictr.org.cn (ChiCTR2000030025)and all patients gave written informed consent. Patients undergoing thyroidectomy with or without neck lymph node dissection between February 2020 to June 2020 were consecutively included in a prospective cohort. Previous thyroid or parathyroid surgery, parathyroid tumor and parathyroid dysfunction, including primary and secondary hyperparathyroidism, were exclusion criteria.

The equipment used in this study was a commercially available, handheld NIR camera (PDE-Neo II; Hamamatsu Photonics). The camera emits infrared light at a wavelength of 760 nm and captures NIR wavelengths of 790–830 nm. Since the light from an LED lamp has little influence on the imaging process, we were able to keep the routine lighting of the operating room and simply move the surgical light away from the surgical field, which reduced the impact of frequent light switching (**Supplementary Figure 1**).

### Surgical Process

**Figure 1:**
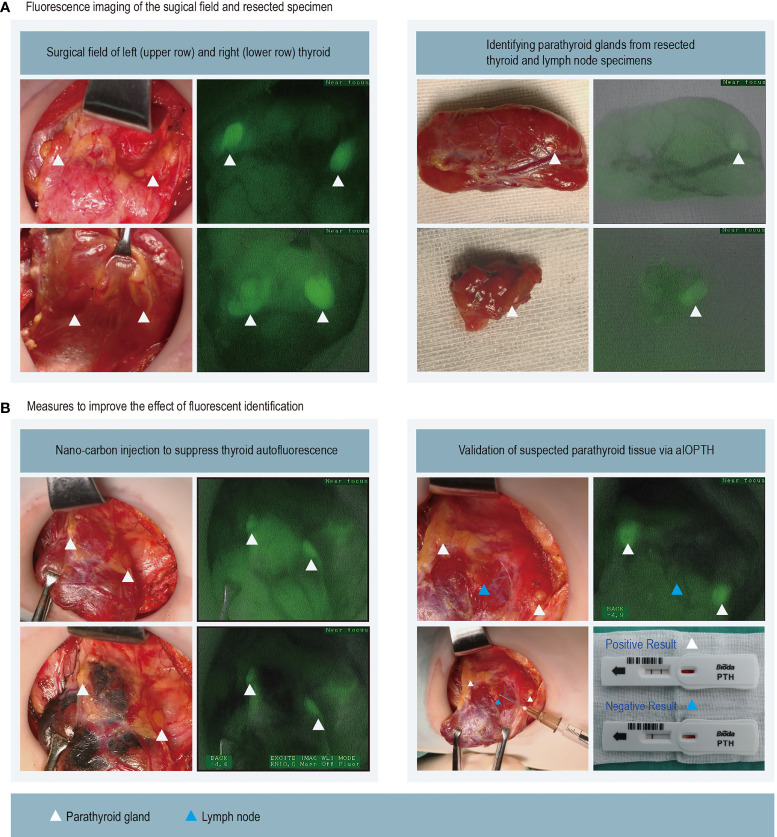
We used fluorescence imaging of the surgical field and resected thyroid and lymph nodes specimens to locate the parathyroid gland. Parathyroid tissues can be highlighted as green in the images **(A)**. Nano-carbon can suppress the fluorescence of the thyroid gland and thus make it easier to recognize parathyroid glands. The lymph node (blue triangle) was visually recognized as the parathyroid gland, but it had weak fluorescence and was finally excluded via aIOPTH **(B)**.

After routine dissection of the band muscles and exposure of the thyroid gland, the first fluorescence imaging was performed. If the thyroid autofluorescence intensity was too strong, then nanocarbon (Chongqing Laimei Pharmaceutical Co) was slowly injected into the thyroid gland until the gland was black-stained. While the surgeon used NIRAF to identify PGs, he also identified PGs through routine visual observation (**Supplementary Video 1**). The fluorescent images using NIRAF to identify the PGs were saved, and the assistant took pictures with a high-resolution camera of the same operative field for subsequent image comparison. The tissues co-identified by vision and NIRAF as PGs were considered as confirmed PGs. If NIRAF revealed PGs in areas that had been visually neglected, it was considered that NIRAF first discovered them. If the judgment of tissue was inconsistent, the measurement of aIOPTH was used for confirmation (**Supplementary Video 2**). The specific method was to puncture the suspicious tissue with a 1 ml syringe containing 0.1 ml saline and then to drip the 0.1 ml saline through the needle onto the reaction area of the test strip and culture for 10 minutes according to the instructions of the parathyroid hormone (PTH) detection kit (Bioda Diagnosis Co) (**Supplementary Figure 2**). If the saline contained PTH, it would combine with PTH antibodies in the reaction zone to form a complex. The strip included the distal control band and the proximal test band. When the liquid containing the complex flowed from the reaction zone to the test band, the complex bound to the anti-PTH antibody in the test band, making it appear red. Furthermore, the detector was used to calculate the PTH level according to the color depth of the band, which ranged from 10 pg/mL to 1,000 pg/mL. A PTH dose greater than 130 pg/mL was regarded as the standard to justify the node as a parathyroid gland (**Figure 1**).

Finally, all tissues not identified as PGs during the procedure were examined pathologically. Among them, nonparathyroid tissues confirmed by aIOPTH were classified into two groups (the visually suspected parathyroid group and the NIRAF suspected parathyroid group) and submitted for pathology separately. The pathologist composed a report for each punctured tissue and carefully searched for the presence of PGs in all tissues.

### Lymph Node Fluorescence Imaging

To observe the fluorescence intensity of the lymph nodes, we separated the specimens of lateral neck lymph node dissection into a high fluorescence intensity group and a low fluorescence intensity group under the fluorescence image and sent them separately for pathological examination. The number of lymph nodes in both groups was collected.

### Data Collection

The following variables were collected during the trial: sex, age, body mass index (BMI; calculated as weight in kilograms divided by height in meters squared), preoperative calcium level, preoperative PTH level, type of surgery, number of patients who used nanocarbon during surgery, number of PGs first identified by NIRAF, number of identified PGs during total thyroidectomy and thyroid lobectomy separately, number of autotransplanted PGs, number of inadvertently resected PGs, PTH in fine-needle aspiration washout fluids from the PG, final diagnosis, postoperative hypocalcemia at postoperative day 1 or 2, nadir of postoperative calcium (considered in the normal range when it was >8.0 mg/dL; to convert to mmol/L, multiply by 0.25), PTH level at postoperative day 1 (considered in the normal range when it was ≥15 pg/mL; to convert to ng/L, multiply by 1.0), number of lymph nodes in lateral neck lymph nodes dissection and hypocalcemia at postoperative month 1.

Because the fluorescence intensity in the image could not be quantified in the imaging system, we used ImageJ software (National Institutes of Health, Bethesda, MD) to measure the fluorescence intensity of different tissues after surgery. First, we confirmed the type of certain tissue in the image and then used the ROI tool in ImageJ software to circle the boundaries of different tissues in the image and measure the average fluorescence intensity of the tissue within the boundary. When multiple identical tissues existed in one image, these tissues were continuously circled, and then the overall average fluorescence intensity was measured.

### Quality Control of the Surgical Process and Data Collection

All operations were performed by the same high-volume surgeon and his assistant, who completed over 800 thyroidectomies a year together. Opinions for visual identification of the PGs were obtained from the surgeon. Before the end of the operation, the assistant checked to ensure that the necessary images and photos were correctly retained and that all specimens sent for pathological examination were correctly labeled.

The image analysis process was completed by the surgeon and his assistant after obtaining the postoperative pathological report. The first step was to compare the image with the operation record and pathological report and then to mark the corresponding tissue type in the fluorescent image. The confirmation of the thyroid and lymph nodes was based on the pathological report, confirmation of the PGs was obtained from aIOPTH results and the pathology report, and adipose tissue confirmation came from the pathological report and the judgment of the surgeon. The process of circle tissue boundary in ImageJ was completed by the surgeon and assistant. To reduce the error in the circle selection process, the arithmetic mean of the measurement results from both of them was used as the final result.

### Statistical Analysis

Non-Gaussian distribution continuous variables (BMI, Preoperative calcium, Parathyroid hormone) are reported using medians and interquartile ranges (IQRs); autofluorescence intensity of tissues was tested to be normally distributed and thus reported as Mean±SD; categorical variables are reported using counts, percentages, and 95% CIs. Pearson’s chi-square analysis was performed to compare the sensitivity and accuracy of NIRAF with vision in identifying parathyroid glands. The sensitivity was calculated by dividing the detected PGs by the final confirmed PGs. The accuracy was calculated by dividing the number of correctly judged tissues by the number of all suspicious tissues in this study. A paired-sample t-test was performed to compare the fluorescence intensity of PGs and other tissues separately. All analyses were 2-tailed, with p ≤ 0.05 set as the criterion for statistical significance. All data were analyzed using SPSS statistical software, version 24.0 (SPSS, Inc.).

## Results

Overall, 250 patients were enrolled in this study, 122 patients used nanocarbon tracers due to strong thyroid autofluorescence during initial imaging, of whom 12 patients had contaminated the surgical field after injecting nanocarbon into the thyroid gland, which made subsequent NIRAF difficult to perform and thus were excluded from the study. The remaining 238 patients completed the trial; 145 patients underwent thyroid lobectomy, and 93 patients underwent total thyroidectomy. Most of them (198 of 238 patients; 83.2%) were diagnosed with malignant tumors. A total of 186 patients (186 of 238 patients; 78.2%) underwent lymph node dissection. Fifty-seven of 93 patients (61.3%) who underwent total thyroidectomy had 4 PGs confirmed, while 118 of 145 patients (81.4%) who underwent thyroid lobectomy had 2 PGs confirmed. Forty-nine patients (20.6%) developed hypocalcemia on the first or second postoperative day. A total of 234 patients were followed up for postoperative calcium, 2 of whom still had hypocalcemia one month after surgery, but these two patients had recovered in the second follow-up at 3 months (**Table 1**).

**Table 1:** Clinical Characteristic of Participants.

Characteristic	Patients No. (%) (N=238)	
**Preoperative Variables**		
Sex, No. (%)		
Male	54 (22.7)	
Female	184 (77.3)	
Age, median (IQR), y	45.0 (36.0-54.0)	
BMI, median (IQR)	25.4 (22.3-27.4)	
Preoperative calcium, median (IQR), pg/mL	9.20 (8.91-9.56)	
Preoperative parathyroid hormone, median (IQR), pg/mL	33.9 (27.6-43.6)	
**Operative Variables**		
Type of surgery.		
Total thyroidectomy with lymph nodes dissection	89 (37.4)	
Total thyroidectomy without lymph nodes dissection	4 (1.69)	
Thyroid lobectomy with lymph nodes dissection	97 (40.8)	
Thyroid lobectomy without lymph nodes dissection	48 (20.2)	
Patients who used nano-carbon during surgery	110 (46.2)	
Identified parathyroid glands during total thyroidectomy, No.		
1	1 (1.1)	n=93
2	1 (1.1)	
3	34 (36.6)	
4	57 (61.3)	
Identified parathyroid glands during thyroid lobectomy, No.		
0	1 (0.7)	n=145
1	26 (17.9)	
2	118 (81.4)	
Autotransplanted parathyroid glands, No.		
0	215 (90.3)	
1	21 (8.8)	
2	2 (0.8)	
Inadvertently resected parathyroid glands	1 (0.4)	
Parathyroid hormone^a^ in fine-needle aspiration washout fluids of parathyroid, median (IQR), mg/dL	264 (198-367)	
**Postoperative Variables**		
Final diagnosis		
Benign condition	47 (19.7)	
Malignant condition	198 (83.2)	
Postoperative hypocalcemia at postoperative day 1 or 2	49 (20.6)	
Nadir of postoperative calcium, median (IQR), mg/dL	8.54 (8.21-9.07)	
Parathyroid hormone at postoperative day 1, median (IQR), pg/ml	24.6 (17.8-34.5)	
Hypocalcemia at postoperative 1 month	2^b^	

BMI, body mass index (calculated as weight in kilograms divided by height in meters squared); IQR, interquartile range. SI conversion factor: To convert calcium to mmol/L, multiply by 0.25; to convert parathyroid hormone to ng/L, multiply by 1. ^a^Parathyroid hormone was tested by the rapid parathyroid hormone detection based on immune colloidal gold technique. ^b^There were 4 losses in the follow-up period.

Overall, 841 suspicious parathyroid tissues were identified; 490 tissues were co-confirmed by vision and NIRAF as PGs, of which 88 PGs were first identified by NIRAF. A total of 351 tissues showed inconsistency between vision and NIRAF, and fine-needle aspiration was performed. Of the 128 tissues recognized as PGs only by NIRAF, 78 were eventually confirmed. Correspondingly, of 223 tissues only visually recognized, only 27 were confirmed as PGs. The mean PTH in fine-needle aspiration washout fluids of the parathyroid was 264 pg/mL.

**Figure 2:**
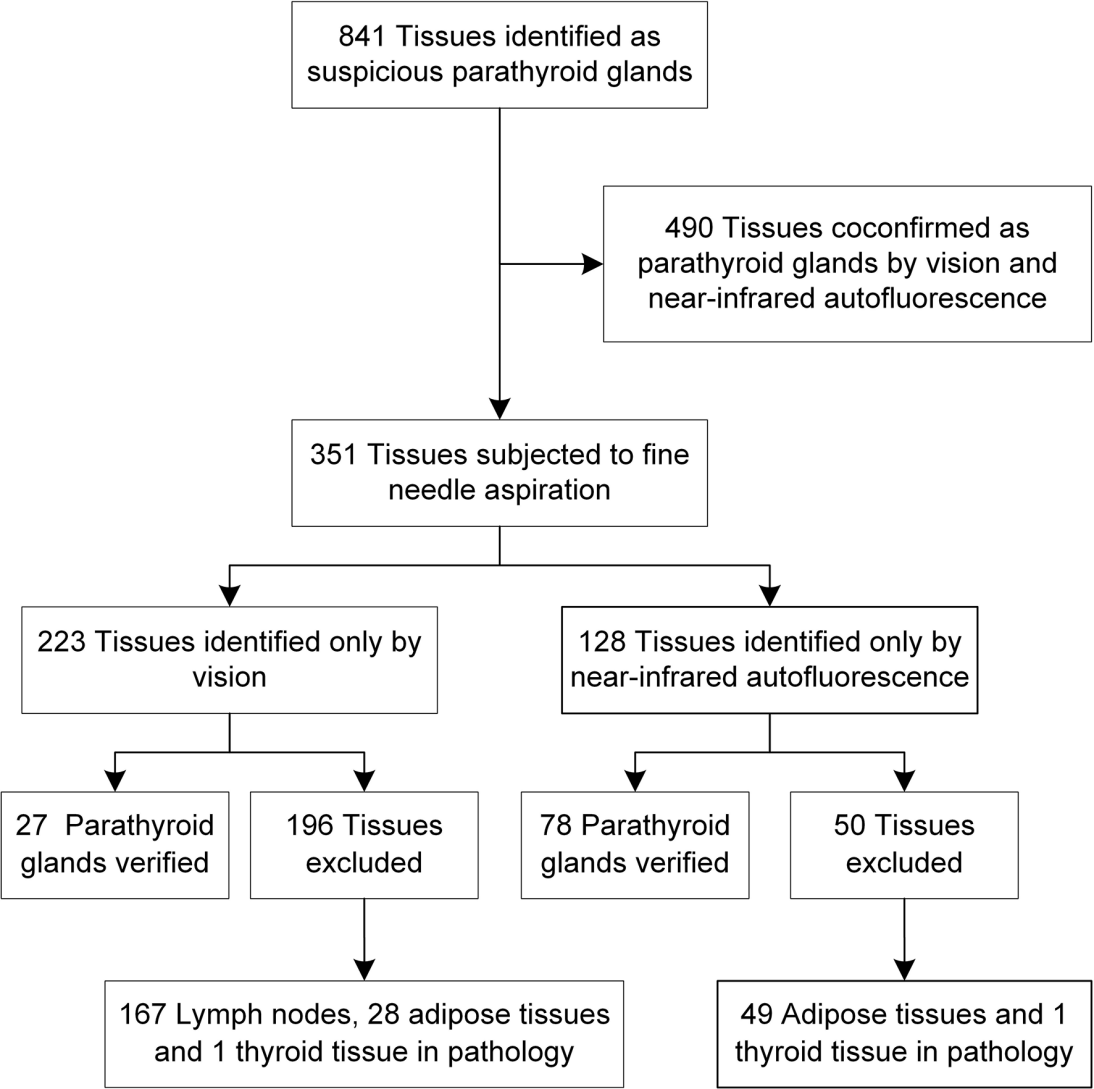
The verification process of 841 suspicious parathyroid tissues.

Pathological examinations were performed on all the resected specimens during the surgery, including 246 labeled tissues with aIOPTH lower than 130 pg/ml. Of the 246 tissues, no parathyroid tissue was found, which was consistent with the aIOPTH results. There were 167 lymph nodes, 28 adipose tissues and 1 thyroid tissue among the tissues misidentified as PGs visually. Forty-nine tissues misidentified by NIRAF were adipose in pathology, and 1 tissue was thyroid (**Figure 2**). An inadvertently resected PG was found in the regular specimens sent for the final pathological examination.

Based on the pathological and aIOPTH results, the sensitivity of NIRAF to detect PGs (568 of 596 PGs; 95.30%) was significantly higher than that of vision (517 of 596 PGs; 86.74%, *P*<.001). Out of 841 suspected parathyroid tissues, there were 595 PGs, 167 lymph nodes, 77 adipose tissues and 2 thyroid tissues (**Figure 2**). The accuracy rate of NIRAF (764 of 841 tissues; 90.84%) was significantly higher than that of vision (567 of 841 tissues; 67.42%, *P*<.001) (**Supplementary Table 1**).

**Figure 3:**
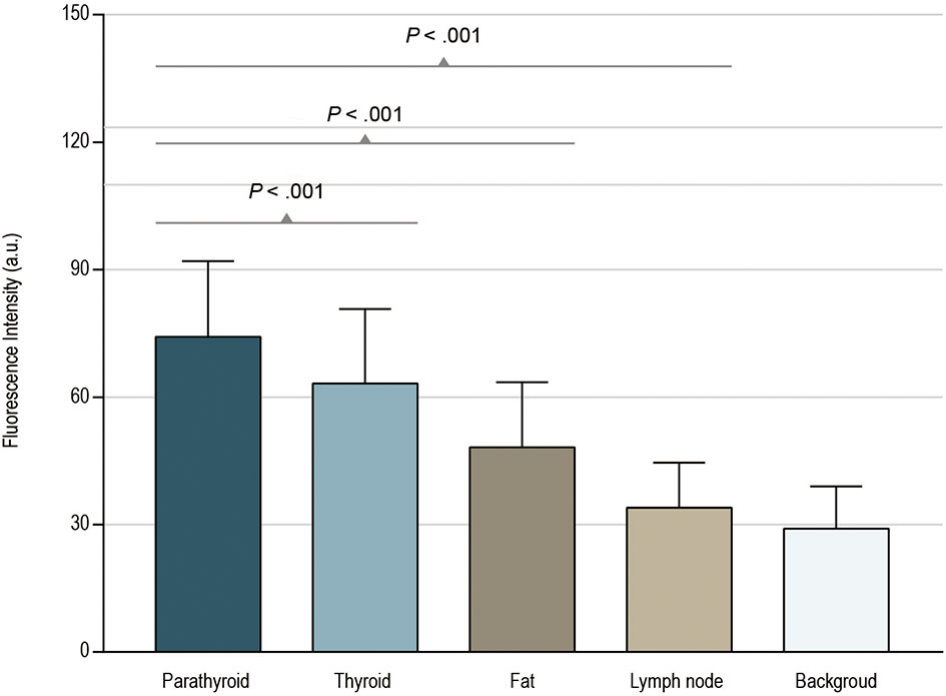
Paired-samples t-test of fluorescence intensity in different tissues.

Combined with the final parathyroid identification results, a retrospective analysis was performed on the selected 331 fluorescent images. The mean fluorescence intensity was 74.19 ± 17.82 for parathyroid tissue in 331 images, 63.24 ± 17.53 for thyroid tissue in 331 images, 48.22 ± 15.31 for adipose tissue in 266 images, and 33.97 ± 10.64 for lymph nodes in 167 images (**Figure 3**). The fluorescence intensity of the parathyroid was significantly higher than that of other tissues in paired samples. The mean increase in parathyroid fluorescence intensity was 10.96 (95% CI, 9.18-12.73) with thyroid tissue (*P*<.001), 26.49 (95% CI, 24.63-28.36) with adipose tissue (*P*<.001), and 40.30 (95% CI, 38.00-15.02) with lymph node tissue (*P*<.001) (**Supplementary Table 2**).

**Figure 4:**
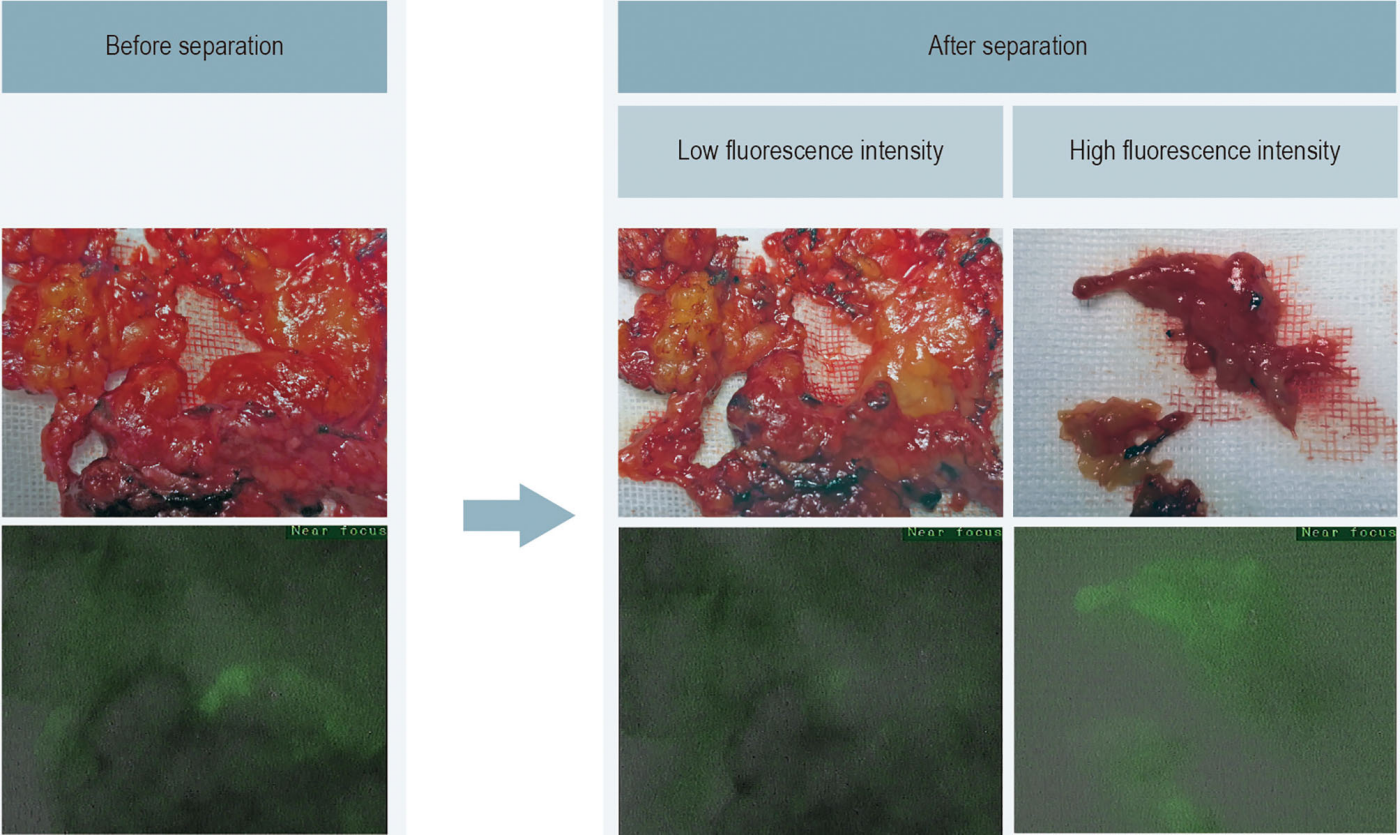
Fluorescence imaging of lateral neck lymph node dissection specimens.

The lateral neck dissection specimens of 22 patients were sorted according to the autofluorescence intensity. The final pathological results showed that there were 536 lymph nodes in the tissues with low fluorescence intensity. No lymph nodes were found in the tissues with high fluorescence intensity (**Figure 4**).

## Discussion

To date, the highest quality of evidence on the effect of NIRAF comes from a multicenter randomized controlled study published by Benmiloud et al. (4), which showed that the rate of postoperative low calcium was significantly lower than that of the control group (from 26% to 11%). The rate of postoperative hypocalcemia after using NIRAF in our study was 20.6%. Our study included a higher proportion of patients with malignant tumors (83.2% *vs* 25.6%), and lymph node dissection was performed in most patients (186 of 238 patients, 78%), which led to a higher rate of postoperative hypocalcemia. However, the mean number of PGs found on each side of the thyroid in their study was 1.61 (390 PGs of 242 patients), compared to 1.80 (595 PGs of 331 patients) in our study. We found more PGs benefiting from the use of nanocarbon but the incidence of postoperative hypocalcemia increased, which indicated that the scope of the operation and protection of the parathyroid blood supply also had a crucial impact (14, 15). The effect of NIRAF is limited to the identification of PGs. Therefore, in evaluating the effect of this technique, the number of identified PGs during surgery is a more direct variable.

The innovation of this study is the introduction of aIOPTH as a confirmatory method for PGs. The application of aIOPTH may provide an alternative to frozen section examination, dodging the injurious effect of the bioptic process and achieving similar or even more accurate results (16–18). However, the application of aIOPTH is not widespread, many endocrine surgeons are not familiar with this technology, and traditional PTH detection methods take much time (19). The rapid PTH detection method used in this study only takes approximately 10 minutes. In actual use, the test strip can turn red in an even shorter time with a positive result. When the cut-off value is 130 pg/mL, this method has proven to be highly consistent with pathological results in identifying PGs (20, 21). It has already been widely used in China and written in the 2018 perioperative parathyroid function protection guidelines for thyroid surgery by the Chinese Thyroid Association. Comparing the pathological results, aIOPTH achieved 100% specificity in our study.

To date, most studies have used visual recognition to confirm PGs due to ethical limitations. The results showed that NIRAF had extremely high sensitivity in identifying PGs (from 76% to 100%) (5, 8, 22). Most studies found that NIRAF could detect PGs before vision in nearly two-thirds of cases (4, 8, 23). Our study concluded that the sensitivity of NIRAF for identifying PGs was 95% by combining aIOPTH results and pathological results, and in the inconsistent cases between vision and NIRAF, the accuracy of NIRAF was 90.84%, while the accuracy of vision was only 67%, indicating that NIRAF was more trustworthy. Among the co-confirmed PGs, 88 PGs were first detected by NIRAF, and 78 PGs were found only by NIRAF. Therefore, of the 596 PGs found in the trial, the surgeon benefited from NIRAF in identifying 166 (28%) PGs.

By comparing the fluorescence intensity of different tissues, we found that the autofluorescence intensity of the thyroid gland was closest to that of the PG (74.19 ± 17.82 *vs* 63.24 ± 17.53). This is concordant with a study by Falco et al. (22). The phenomenon that the autofluorescence intensity of the thyroid gland occasionally approaches or even exceeds the PG during surgery often affects the detection of PGs, and we suppressed the autofluorescence of the thyroid by injecting nanocarbon into the thyroid gland, thereby improving the fluorescence contrast of the PGs.

Of note, there was a significant difference in the autofluorescence intensity of the parathyroid and lymph nodes (74.19 ± 17.82 *vs* 33.97 ± 10.64), which is consistent with the result from Shinden et al. (24). Therefore, no lymph nodes were found in the 50 NIRAF false-positive tissues. Furthermore, we used NIRAF to sort out the tissues with no obvious fluorescence in lateral neck lymph node dissection, in which 536 lymph nodes were found pathologically. Meanwhile, no lymph nodes were found in the tissues with strong fluorescence intensity. We conclude from this result that the fluorescence intensity of lymph nodes is pretty weak compared to that of PGs. Lymph nodes are often misidentified as PGs during thyroid surgery. Of the 196 visual false-positive tissues in this study, 167 were lymph nodes. The surgical strategy of retaining all tissues with strong fluorescence intensity will help ensure a thorough dissection of lymph nodes, thereby reducing the tumor residue caused by misidentifying lymph nodes as PGs.

A total of 351 fine-needle aspirations were performed in 238 patients. According to the final result, the tissues with strong fluorescence intensity are mostly parathyroid and adipose tissues, thyroid tissues appear only in rare cases, which can be retained without further testing. During the trial, even if we identified a PG in a certain area, we still tried to find other suspicious parathyroid tissues. It is not necessary to do so in clinical applications, so the frequency of aIOPTH can be largely reduced, thereby shortening the operation time. The use of NIRAF requires only a small amount of time to remove the surgical lamp. Therefore, the use of NIRAF and aIOPTH technology in thyroid surgery will not significantly increase the duration of surgery.

To date, NIRAF has satisfied but not perfect sensitivity and specificity in published studies (10, 25). However, the strong fluorescence of thyroid sometimes may cover the fluorescence of PGs and thus decrease the sensitivity of NIRAF. The use of nanocarbon can solve the problem. Meanwhile, aIOPTH can separate false-positive tissues with high fluorescence from PGs and enhance the specificity. All these measures are time-saving and easy to implement. These methods along with NIRAF form a perfect complement in thyroid surgery, which can effectively find atypical PGs and prevent them from being inadvertently resected.

This case series study, despite its careful experimental design, strict quality control, and a large number of patients, is still not enough to show that this new surgical strategy can improve the number of identified PGs. Therefore, we plan to conduct a further randomized controlled study to illustrate this issue. Another drawback is that we did not puncture the PGs identified by both vision and NIRAF, which is mainly based on the purpose of reducing parathyroid damage. Although fine-needle biopsy poses minimal damage to the tissue, there is still a report that such puncture may cause secondary lesions of the PGs (26).

## Conclusions

In conclusion, we evaluated the effect of NIRAF in identifying PGs in thyroid surgery through a large-sample case series. Using NIRAF in thyroid surgery can effectively assist surgeons in identifying PGs and distinguishing lymph nodes when lymph node dissection is performed. Measuring aIOPTH was an ideal objective method to evaluate the effect of NIRAF. It may work with nanocarbon as complements to NIRAF and thus improves the sensitivity and specificity.

## Data Availability Statement

The original contributions presented in the study are included in the article/**Supplementary Material**. Further inquiries can be directed to the corresponding author.

## Ethics Statement

The studies involving human participants were reviewed and approved by The institutional review board of the First Affiliated Hospital of Zhengzhou University. The patients/participants provided their written informed consent to participate in this study.

## Author Contributions

Conception and design: ZL and D-tY. Administrative support: ZL and D-tY. Provision of study materials or patients: ZL and D-tY. Collection and assembly of data: ZL, R-sM, J-lJ, TW, and D-hZ. Data analysis and interpretation: ZL and J-lJ. All authors contributed to the article and approved the submitted version.

## Funding

This work was supported by the following funds: Young Talents Promotion Program of Henan Science and Technology Department (2020HYTP055), Major Scientific Research Projects of Traditional Chinese Medicine in Henan Province (No.20-21ZYZD14), and Cultivation of Young and Middle-aged Health Science and Technology Innovation Leading Talents in Henan Province (YXKC2020015).

## Conflict of Interest

The authors declare that the research was conducted in the absence of any commercial or financial relationships that could be construed as a potential conflict of interest.

## Publisher’s Note

All claims expressed in this article are solely those of the authors and do not necessarily represent those of their affiliated organizations, or those of the publisher, the editors and the reviewers. Any product that may be evaluated in this article, or claim that may be made by its manufacturer, is not guaranteed or endorsed by the publisher.

## Supplementary Material

Supplementary Table 1 Comparing the sensitivity and accuracy of NIRAF with vision in identifying parathyroid glands. PGs parathyroid glands; ^a^The sensitivity of NIRAF was significantly higher than vision in 596 parathyroid glands, *P*<.001. ^b^The accuracy of NIRAF was significantly higher than vision in 841 suspected parathyroid tissues. *P*<.001

Supplementary Table 2 Paired-samples t-test of fluorescence intensity in different tissues.

Supplementary Figure 1 Fluorescence imaging device and operating environment. The operating room illuminated by LED light sources does not need to turn off the light, only needs to remove the operating lamp, but the operating room illuminated by a halogen lamp needs to turn off the light source. During the NIRAF imaging process, it is necessary to avoid the camera shooting of the surgery field, which will cause the flicker of the fluorescent image. The electrocautery should be used with caution during the operation to reduce the generation of eschar, which has extremely strong autofluorescence under fluorescent imaging.

Supplementary Figure 2 Test strip and parathyroid hormone detector for measuring intraoperative parathyroid hormone in fine-needle aspiration biopsy washings. **(A)** Control line **(B)** Test line **(C)** Reaction zone. The test line requires a 10-minute incubation time to reach a stable final result, but in most cases, the test line can turn red in a shorter time with a positive result. Parathyroid hormone detector is not needed if exact values are not required

Supplementary Video 1 Intraoperative near-infrared autofluorescence imaging of parathyroid glands. The NIRAF probe is wrapped with a sterile lens cover, and the surgeon holds the NIRAF probe with one hand to perform fluorescence imaging of the surgical field. The operating room lighting can be kept after removing the surgical lamp from the surgical field.

Supplementary Video 2 Measuring intraoperative parathyroid hormone in fine-needle aspiration biopsy washings of the suspicious parathyroid gland. The suspicious tissue with strong fluorescence intensity was punctured with a 1 ml syringe containing 0.1 ml saline and then drip the 0.1 ml saline through the needle onto the reaction area of the test strip.
