# Supplementary material for: Evaluation of Autofluorescence in Identifying Parathyroid Glands by Measuring Parathyroid Hormone in Fine-Needle Biopsy Washings
Source: Front Endocrinol (Lausanne). 2022 Jan 21;12:819503. doi: 10.3389/fendo.2021.819503 (PMC8815459; doi:10.3389/fendo.2021.819503)
Supplement: Supplementary Table 1 — Comparing the sensitivity and accuracy of NIRAF with vision in identifying parathyroid glands. PGs parathyroid glands; aThe sensitivity of NIRAF was significantly higher than vision in 596 parathyroid glands, P<.001. bThe accuracy of NIRAF was significantly higher than vision in 841 suspected parathyroid tissues. P<.001 [file Table_1.docx]

**Supplementary Table 1. Comparing the sensitivity and accuracy of NIRAF with vision in identifying parathyroid glands**

| **Method** | Identified PGs No. | Undiscovered PGs No. | **Sensitivity^a^** |
| --- | --- | --- | --- |
| NIRAF | 568 | 28 | 95.30% |
| Vision | 517 | 79 | 86.74% |

| **Method** | Correctly identified tissues No. | Misidentified tissues No. | **Accuracy^b^** |
| --- | --- | --- | --- |
| NIRAF | 764 | 77 | 90.84% |
| Vision | 567 | 274 | 67.42% |

PGs parathyroid glands；

a The sensitivity of NIRAF was significantly higher than vision in 596 parathyroid glands, *P*<.001

b The accuracy of NIRAF was significantly higher than vision in 841 suspected parathyroid tissues. *P*<.001
