# Supplementary material for: Evaluation of Autofluorescence in Identifying Parathyroid Glands by Measuring Parathyroid Hormone in Fine-Needle Biopsy Washings
Source: Front Endocrinol (Lausanne). 2022 Jan 21;12:819503. doi: 10.3389/fendo.2021.819503 (PMC8815459; doi:10.3389/fendo.2021.819503)
Supplement: Supplementary Table 2 — Paired-samples t-test of fluorescence intensity in different tissues. [file Table_2.docx]

**Supplementary Table 2. Paired-samples t-test of fluorescence intensity in different tissues**

|  |  | **Paired fluorescence intensity difference value** | |  |  |
| --- | --- | --- | --- | --- | --- |
| **Paired group** | **Paired No.** | **Mean±SD** | **95%CI** | **t** | ***P* value** |
| Parathyroid-thyroid | 331 | 10.96±16.42 | 9.18-12.73 | 12.14 | <.001 |
| Parathyroid-Fat | 266 | 26.49±15.46 | 24.63-28.36 | 27.94 | <.001 |
| Parathyroid-lymph node | 167 | 40.30±15.02 | 38.00-42.59 | 34.68 | <.001 |
