# Supplementary figures and images for: Evaluation of Autofluorescence in Identifying Parathyroid Glands by Measuring Parathyroid Hormone in Fine-Needle Biopsy Washings
Source: Front Endocrinol (Lausanne). 2022 Jan 21;12:819503. doi: 10.3389/fendo.2021.819503 (PMC8815459; doi:10.3389/fendo.2021.819503)

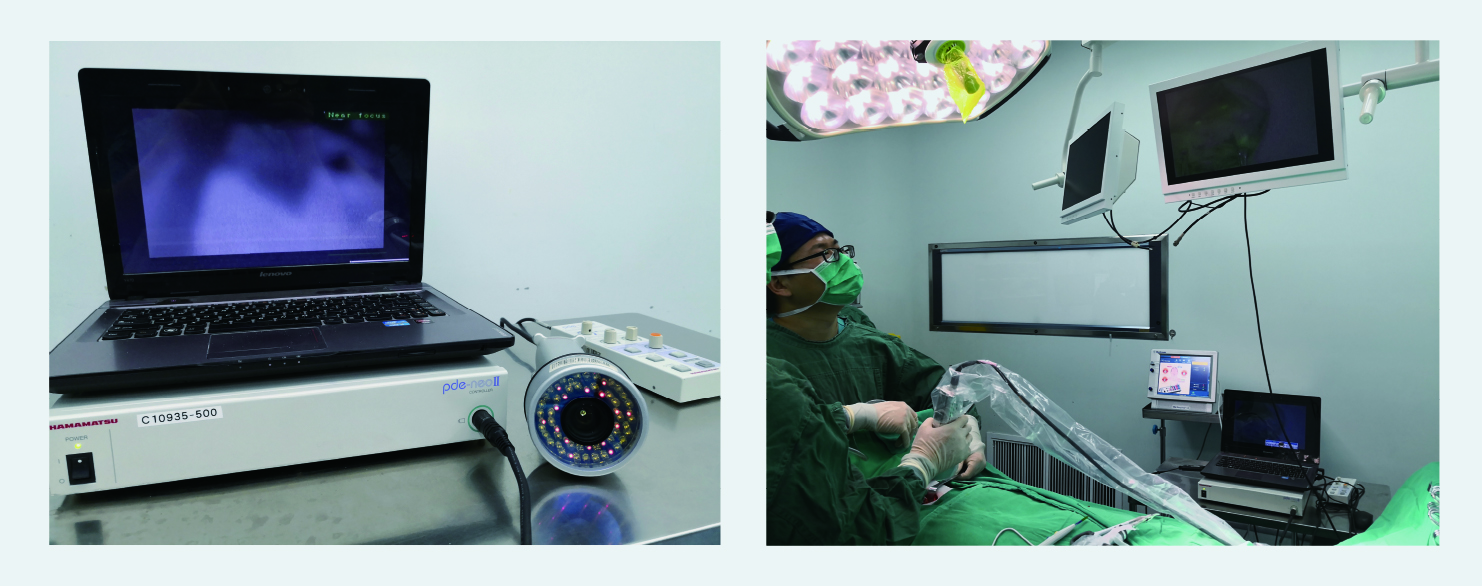

Supplement: Supplementary Figure 1 — Fluorescence imaging device and operating environment. The operating room illuminated by LED light sources does not need to turn off the light, only needs to remove the operating lamp, but the operating room illuminated by a halogen lamp needs to turn off the light source. During the NIRAF imaging process, it is necessary to avoid the camera shooting of the surgery field, which will cause the flicker of the fluorescent image. The electrocautery should be used with caution during the operation to reduce the generation of eschar, which has extremely strong autofluorescence under fluorescent imaging. [file Image_1.jpeg]

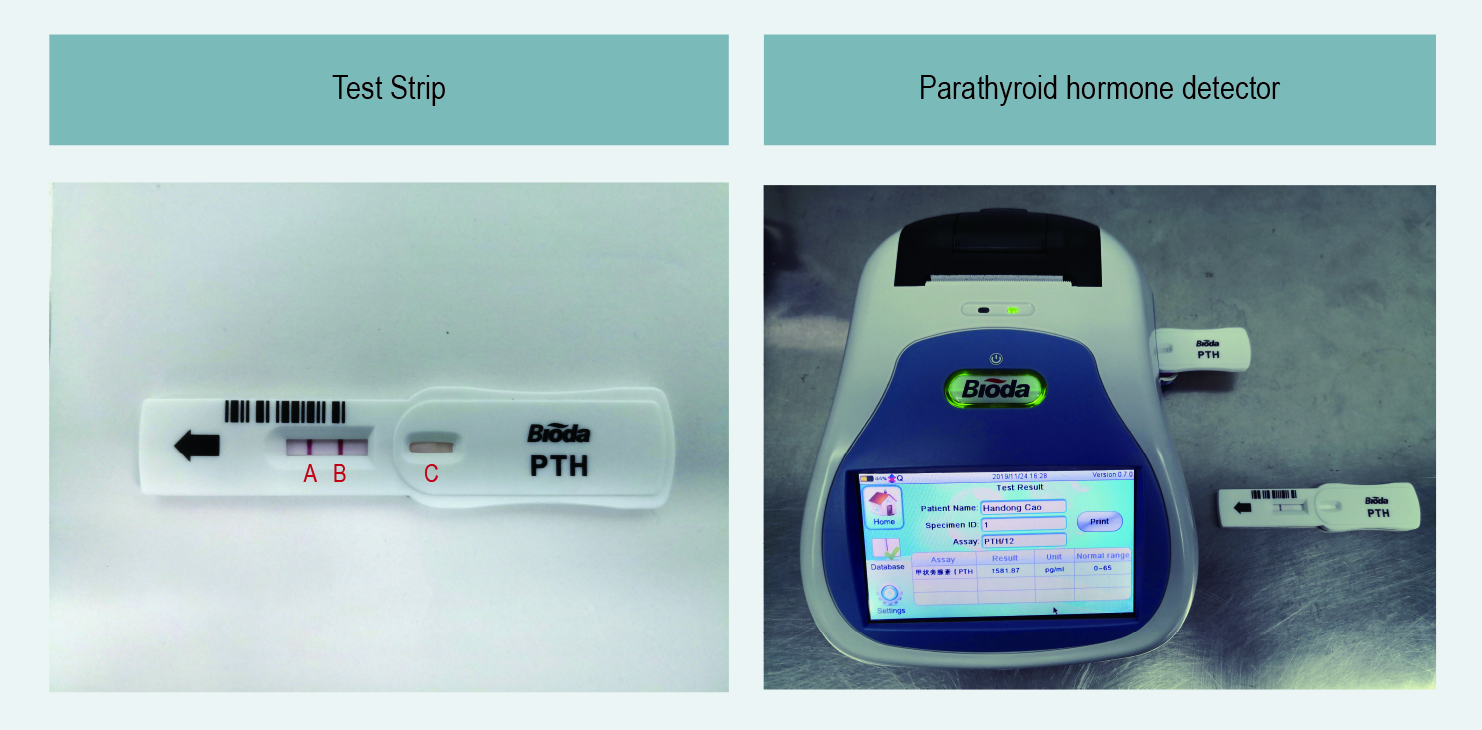

Supplement: Supplementary Figure 2 — Test strip and parathyroid hormone detector for measuring intraoperative parathyroid hormone in fine-needle aspiration biopsy washings. (A) Control line (B) Test line (C) Reaction zone. The test line requires a 10-minute incubation time to reach a stable final result, but in most cases, the test line can turn red in a shorter time with a positive result. Parathyroid hormone detector is not needed if exact values are not required [file Image_2.jpeg]
